# Two-dimensional quantitative near-field phase imaging using square and hexagonal interference devices

**DOI:** 10.1515/nanoph-2022-0215

**Published:** 2022-08-26

**Authors:** Petr Dvořák, Pavel Klok, Michal Kvapil, Martin Hrtoň, Petr Bouchal, Jan Krpenský, Vlastimil Křápek, Tomáš Šikola

**Affiliations:** Institute of Physical Engineering, Brno University of Technology, Technická 2, 616 69 Brno, Czech Republic; CEITEC Brno University of Technology, Purkyňova 123, 612 00 Brno, Czech Republic

**Keywords:** interference nanostructures, near-field, phase imaging, SNOM, SPP waves

## Abstract

We demonstrate the formation of the near field with non-trivial phase distribution using surface plasmon interference devices, and experimental quantitative imaging of that phase with near-field phase microscopy. The phase distribution formed with a single device can be controlled by the polarization of the external illumination and the area of the device assigned to the object wave. A comparison of the experimental data to a numerical electromagnetic model and an analytical model assigns the origin of the near-field phase to the out-of-plane electric component of surface plasmon polaritons, and also verifies the predictive power of the models. We demonstrate a formation of near-field plane waves with different propagation directions on a single device, or even simultaneously at distinct areas of a single device. Our findings open the way to the imaging and tomography of phase objects in the near field.

## 1 Introduction

Surface plasmon polaritons (SPPs) are surface electromagnetic waves suitable for two-dimensional (2D) on-chip optics [1, 2]. They are not restricted by the diffraction limit and can be thus used for the miniaturization of optical devices [3–6]. The electromagnetic near field (NF) of SPP propagating along a dielectric-metal interface has been thoroughly studied [7, 8]. This field in turn can probe the interface and can be used for optical imaging of objects on the interface [1, 9, 10]. Typical characterization of NF includes its intensity [11, 12]. However, the full beauty of the field is revealed with its phase [13–15], which is considerably harder to retrieve [16, 17]. In this paper, we demonstrate digitally post-processed NF phase distribution formed by the SPP standing waves that emerged from surface plasmon interference devices (SPID) consisting of square or hexagonal slits. The NF patterns can be controlled via the geometry of slits and optical parameters of illumination, mainly polarization.

In the far-field approach, phase imaging continuously develops. The phase images are traditionally provided by holographic systems that evolved from laser-based interferometers to more sophisticated holographic microscopes [18], allowing the illumination of arbitrary coherence [19–21]. Holography is popular for contrasting the weakly scattering samples and providing quantitative phase information. Quantitative phase imaging allows observation of minute changes of the dry mass in live biological cells [22] or studying complex responses of plasmonic nanostructures [23, 24]. The holographic systems also benefit from the recent development of new optical components allowing polarization-selective light-control [25] or better integration [26]. Beyond the holography, the phase can be extracted using computational imaging that allows lensless on-chip microscopy [27], optical ptychography [28], or phase reconstruction from non-interferometric measurement using the transport of intensity equation [29] or Kramers-Kronig relations [30]. Near-field phase imaging with subwavelength resolution would represent a natural extension of the large family of far-field methods. However, the methods for the phase characterization of the near field are less developed.

Available methods for NF characterization are based on scanning probe microscopy (SPM), in particular on the scanning near-field optical microscopy (SNOM), which simultaneously characterizes the topography of the scanned area [31]. A scattering-type SNOM (s-SNOM) for quantitative phase imaging with high lateral resolution (around 20 nm) was developed [32, 33]. This technique works primarily in the near- and mid-infrared spectral range, as the intense far-field background signal caused by the light scattered (or reflected) from the sample surface makes it difficult to correctly interpret s-SNOM images [34, 35], making the s-SNOM thus largely insensitive to the formed patterns of the investigated objects [12, 36]. However, recent works show that it is possible to use the s-SNOM across the visible range for near-field phase distribution [37, 38]. An aperture-type SNOM (a-SNOM) probes possess a subwavelength aperture at the probe tip and, unlike s-SNOM, allows to obtain near-field images of both electric and magnetic fields with roughly equal sensitivity [39]. Aperture-type SNOM (a-SNOM) with a heterodyne configuration can be therefore easily used for NF phase imaging in the visible spectral range [40, 41]. This approach led to numerous interesting studies in 2D nano-optics, e.g., measurement of the phase velocity of SPP wave or quantitative determination of magnetic components of the NF [42, 43]. However, the heterodyne configuration requires a comparison of the collected optical signal with a reference wave in an external device. Therefore, it is not suitable for on-chip applications [44].

Recently, we have developed two-dimensional quantitative near-field phase microscopy (2D QN-FPM), an a-SNOM method for quantitative NF phase imaging with the holography step performed fully in the near field [45]. This method overcomes the need for the external reference wave and is, therefore, suitable for on-chip integration. The only far-field steps involved in the current implementation of 2D QN-FPM are the excitation and the collection. We have demonstrated the performance of 2D QN-FPM in the phase imaging of propagating and standing SPP waves.

As mentioned above, we present a comprehensive quantitative NF phase imaging of interference patterns formed by the linear SPP sources. Our experimental results are supported by finite-difference time-domain (FDTD) numerical calculations and an analytical model. We demonstrate the possibility of preparing the waves of distinct propagation directions on the same device in a controllable way by specifying the polarization of the external illumination and the state of the spatial light modulator.

## 2 Methods and materials

**Figure 1:**
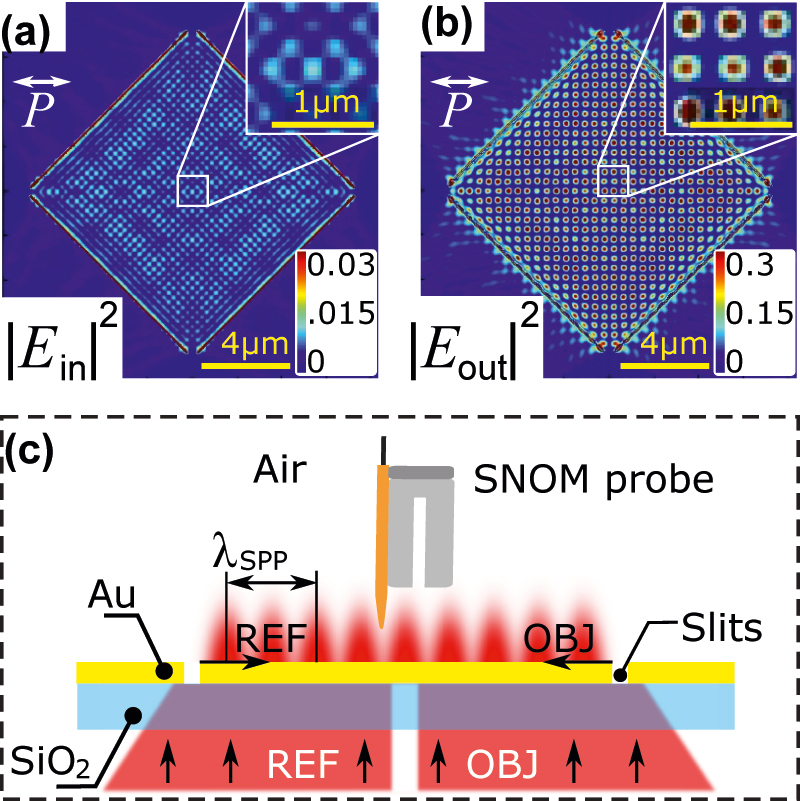
Numerical calculations of near field and scheme of the measuring method. (a) and (b) Calculated distribution of the normalized near-field intensity on the square SPID: (a) in-plane electric and (b) out-of-plane electric component. Polarization of the illumination is indicated with the white arrow. Insets show details of the field distribution in the centre of the SPID. (c) A scheme of the SPID with the illumination wave, the near-field object (OBJ) and reference (REF) waves, and an SNOM probe.

The near field for phase imaging is formed using a so-called surface plasmon interference device (SPID). SPID consists of an opaque gold film deposited on a transparent glass substrate. Subwavelength slits fabricated in gold by focused ion beam (FIB) milling (more details included in Appendix A) support propagation of SPP but absorb far-field electromagnetic waves. When illuminated by a plane wave from one side (bottom), such slits serve as a linear source of SPP on the opposite (top) interface of the gold film [46]. With a specific arrangement of slits, an NF interference pattern can be formed on top of SPID. An example of such a pattern obtained by FDTD numerical simulations for a square SPID (four slits arranged into a square forming the interference of four SPP waves) is shown in Figure 1(a) and (b). The two panels correspond to the in-plane and out-of-plane components of the electric NF. The shape of the interference pattern is characteristic of the polarization of NF and can be used to determine the contribution of each polarization to the resulting image. The experimental setup utilized in this work is sensitive mainly to the out-of-plane component, in agreement with our previous studies [47]. The waves can be thus treated as scalar.

The intensity of NF on SPID is characterized by an a-SNOM. A scheme of the full experiment including the SPID chip, external illumination, and SNOM detection, is shown in Figure 1(c). The phase of NF is reconstructed using 2D QN-FPM developed and published by our research group [45], which is based on phase-shifting digital holography (PSDH). In this method, the object (OBJ) wave whose phase is to be determined interferes with a reference (REF) wave formed on the same SPID. By applying several specific phase shifts to the reference wave, recording the intensity of the resulting interference pattern for each phase shift, and digital post-processing, we can reconstruct the NF phase distribution. Digital post-processing is described in more detail in Appendix A.1. To introduce the phase shift to the REF wave, we utilize a spatial light modulator (SLM) that spatially modifies the phase of the illumination plane wave. The part of the wavefront illuminating the slits forming the REF wave has a phase modified by the SLM, while for the remaining wavefront illuminating the slits forming the OBJ wave, the phase is unchanged. The schematic of the experimental setup is shown in Appendix A.5 (see Figure 9).

## 3 Results

**Figure 2:**
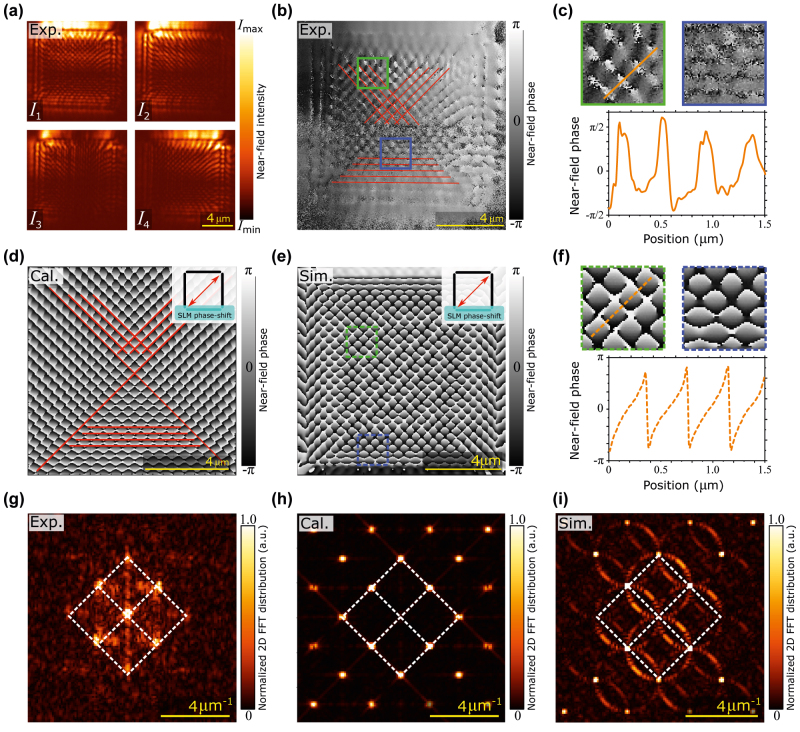
Near-field phase distribution on square SPID. Square SPID with left, upper, and right slit producing OBJ wave and bottom slit producing REF wave: (a) Near-field intensity recorded by SNOM for four phase shifts of the REF wave. (b) Experimentally obtained phase distribution. (c) Details from (b) and linear phase profile. Corresponding areas in (b) and (c) have colour-matched frames; the linear profile is taken along the orange line. (d) Analytical model and (e) FDTD numerical simulations of the phase distribution (f) Details from (e) and linear phase profile. Inset in (d) and (e) shows an arrangement of the slits with the turquoise patch representing the area with a modified phase serving as the source of the REF wave and the red double-sided arrow showing the direction of polarization. (g)–(i) FT of (b)–(e), respectively.

Figure 2(a) shows experimentally obtained intensity of SPP interference patterns for a square SPID where the three slits served as the source of the OBJ wave, and only the bottom slit produced the REF wave. The four panels *I*_1_ to *I*_4_ correspond to four values of the phase shift imposed on the REF wave. Figure 2(b) depicts the 2D distribution of the NF phase reconstructed from the intensities *I*_1_ to *I*_4_. In Figure 2(c), we show in more detail the experimental phase in two specific regions together with a linear profile of the phase. It is noticeable that the linear sawtooth phase profile does not span the full range from −*π* to +*π*. This is attributed to the effect of the background noise, as discussed in more detail in Ref. [45]. Experimentally obtained phase distribution is compared to an analytical model (Figure 2(d)) and to a numerical FDTD simulation (Figure 2(e)). There is a good agreement between the phase distribution obtained by all approaches, as evidenced by both the linear profiles and spatial distributions shown in Figure 2(c) and (f) for the experiment and the numerical simulations, respectively.

Further insight into the reconstructed phase patterns is obtained from Fourier transforms (FT) of the phase maps shown in Figure 2(g)–(i). Again, a good agreement of all three patterns is observed. The lowest periodicity observed in the patterns reads (298 ± 7) nm and corresponds very well to half of the SPP wavelength, confirming the assignment of the observed field to SPP. A comparison of the experimental pattern to the calculated patterns for the in-plane and out-of-plane electric components (not shown) also proves that the image is formed by the out-of-plane component in agreement with our previous observations [47]. Finally, a weak horizontal-vertical asymmetry is related to the apparent asymmetry of the U-shape source of the OBJ wave.

**Figure 3:**
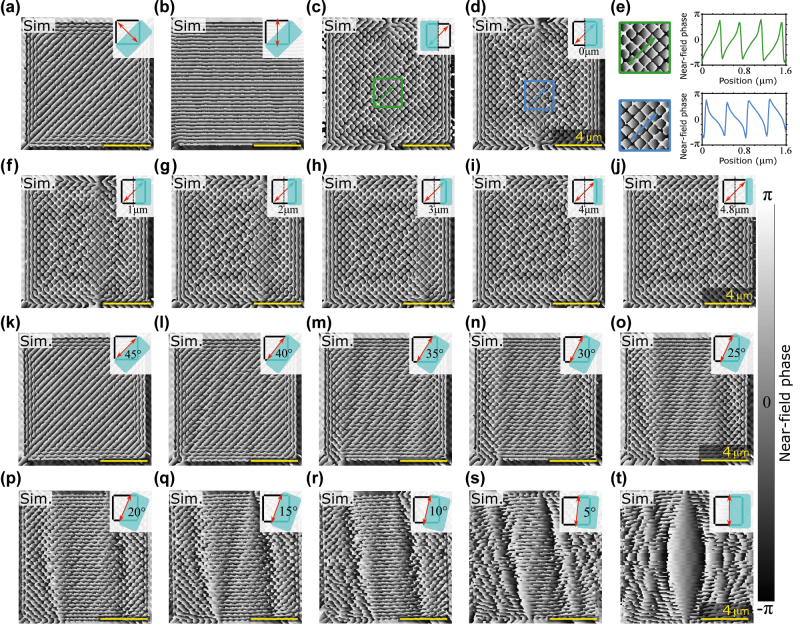
Numerical calculations of near-field phase distribution on square SPID. FDTD simulations of phase formed on a square SPID for different OBJ-REF divisions and polarizations. (a)–(d) High-symmetry examples. (e) Details from (c) and (d) with linear phase profiles. (f)–(j) Narrowing the REF wave region. (k)–(t) Rotating both the polarization and REF wave region in five-degree steps. Insets represent square arrangements of slits with the turquoise patch representing the REF wave area and the red double-sided arrow representing the illumination polarization.

After confirming the predictive power of the numerical simulations experimentally, we now focus on the extensive study of various phase setups accessible with square SPID by modifying the division of the area into OBJ and REF waves and changing the polarization of the illumination wave. To this end, we employ the FDTD simulations. The calculated phase distributions are shown in Figure 3. The first row shows different combinations of the polarization directions REF-OBJ division. Interestingly, Figure 3(c) and (d) differ only in mirrored OBJ and REF waves, and the phase distribution exhibits the same mirror symmetry. The linear phase profiles from these two cases are compared in Figure 3(e), confirming the mirror symmetry. In other words, within a single SPID, it is possible to prepare a left-propagating or right-propagating wave just by a proper selection of the region for OBJ and REF waves. There is an important consequence of such a finding. Imaging phase objects provides a one-dimensional projection of what is being imaged but lacks the capability of two-dimensional imaging. For this purpose, near-field phase tomography can be used, where the common phase imaging is performed for several directions of the imaging wave and the two-dimensional image is reconstructed using Radon transformation [48]. The formation of SPP waves with different propagation directions on a single SPID is an important step towards realizing phase tomography.

In Figure 3(f)–(j), we show the effect of narrowing the REF wave region, which we can achieve by using SLM. We observe a clear change in the phase pattern on the boundary between the OBJ and REF waves (inside and outside U-like arrangement of OBJ slits). Finally, Figure 3(k)–(t) shows the effect of the illumination polarization. In these cases, we observe a gradual formation of phase bands that resemble the Moiré phenomenon or superlattice patterns [49, 50]. We attribute the existence of this phenomenon to the amplification of the influence of SPP waves propagating in non-perpendicular directions towards the slits.

**Figure 4:**
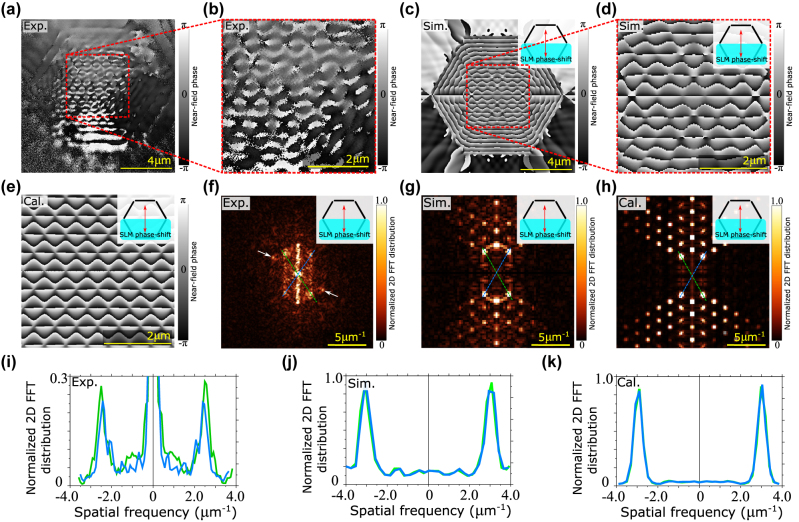
Near-field phase distribution on hexagonal SPID. Hexagonal SPID with three upper slits producing the OBJ wave and three bottom slits producing the REF wave: Phase distribution reconstructed from the experiment (a) with detail (b), numerical calculations (c) with detail (d), and the analytical model (e). (f)–(h) FT of phase distribution shown in (b), (d), (e), respectively. (i)–(k) Profiles along the colour-matched dashed lines in (f)–(h), respectively, show the most frequent modulations from the source image.

After a thorough investigation of square SPIDs, we have focused on hexagonal SPIDs. In Figure 4(a), we present an experimentally obtained 2D distribution of the NF phase for a hexagonal SPID with the excitation polarization and OBJ and REF wave regions shown in the inset of Figure 4(c). The observed phase pattern is well reproduced by both the numerical simulations (Figure 4(c)) and the analytical model (Figure 4(e)), as in the case of the square SPID. The similarity of all three patterns is also confirmed by FT images shown in Figure 4(f)–(h). Two dashed lines (blue and green) connect the most intense peaks in the intensity profile spaced apart plotted in Figure 4(i)–(k) representing the spatial frequency dependence on the normalized FT distribution. The two peaks highlighted by the white arrows in Figure 4(f) indicate an artifact probably caused by illumination inhomogeneity (the slits have different illumination intensity) or slit defects.

**Figure 5:**
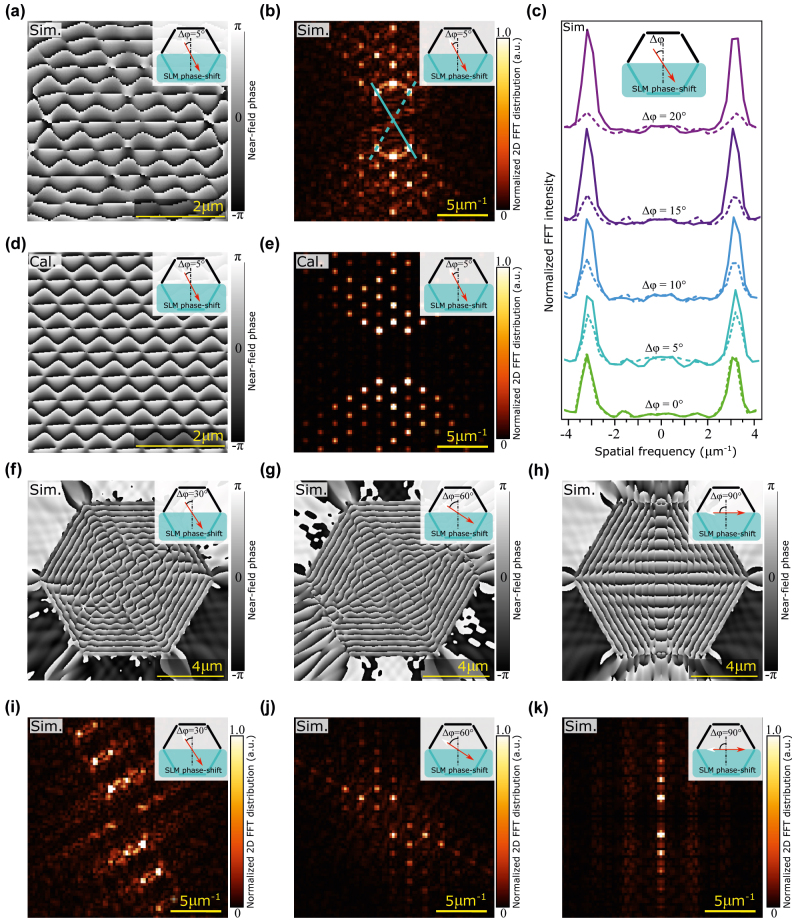
Near-field phase distribution on hexagonal SPID. Hexagonal SPID with three upper slits producing the OBJ wave and bottom three slits producing the REF wave for several polarizations of the illumination wave: FDTD numerical simulation (a) and analytical model (d) with the corresponding FTs (b), (e). Numerical simulations of the phase distribution for several other polarizations of the illuminating wave (f)–(h) with the corresponding FTs (i)–(k). The plot of the most frequent modulations from FTs (c) for several polarizations of the illuminating wave.

In Figure 5, we demonstrate that the phase pattern can be controlled by the polarization of the excitation illumination. Using FT, we extract the most frequent modulations from the source image, which we use for comparison of different polarization angles in Figure 5(c). Clearly, the sensitivity to the polarization is most pronounced in the centre of SPIDs, while close to the slits, the phase patterns are only weakly influenced. This finding is again important for the imaging of phase objects. Close to the slits, the image shall be rather insensitive to the exact polarization of the excitation illumination, eliminating the need for precise control of such polarization. On the other hand, for phase objects at the centre of SPID, it is possible to control the direction of the wave by the polarization of the illumination and thus to perform the phase tomography without the need to change the regions for the OBJ and REF waves.

We will now focus on possible applications of near-field phase imaging. As we have already mentioned, there is a straightforward application in the imaging of phase objects. In microscopy, a phase object is a low-contrast object that only weakly modifies the intensity of a wave transmitted through or reflected by the object. Still, there can be a substantial modification in the phase of the wave, which allows to image the object with improved contrast and even to quantify its optical thickness.

**Figure 6:**
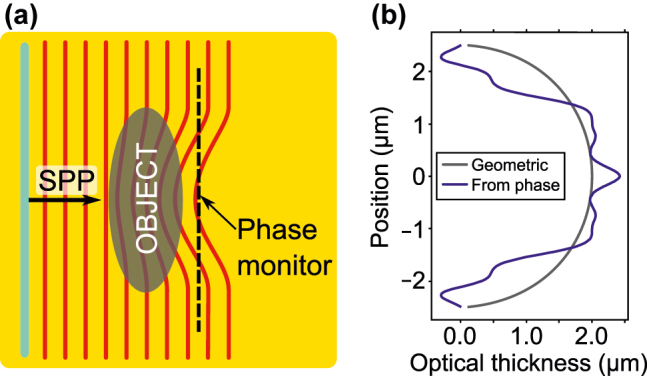
Proof-of-principle of near-field imaging of phase objects. (a) A scheme of a phase object with an incoming SPP “plane wave”; dashed line located 500 nm behind the phase object represents a location where the phase delay is evaluated. (b) The geometric thickness of the phase object (black) compared to the thickness determined from the phase delay (blue).

**Figure 9:**
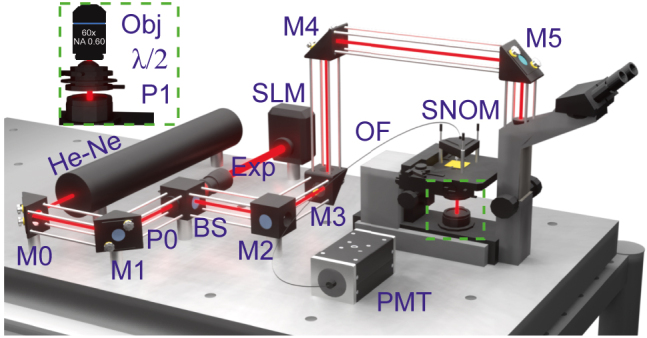
Experimental setup. Experimental setup: (He–Ne) helium-neon laser, (M0–M5) dielectric mirrors, (P0, P1) linear polarizers, (BS) beamsplitter, (Exp) beam expander, (SNOM) scanning near-field optical microscope, (λ/2) half-wave plate, (Obj) 60× objective (NA = 0.6), (OF) optical fibre, (PMT) photomultiplier tube.

While the experimental demonstration of the imaging of phase objects is beyond the scope of this work, we still provide a simple proof-of-principle based on electromagnetic simulations. We consider an SPID with only a single slit (with a length of 10 µm) launching an SPP “plane wave”. The phase object is formed by the elliptic cylinder with a refractive index of 1.1, a height of 100 nm, and diameters of 2 µm and 5 µm, where its shorter diameter is parallel with the propagation direction of the SPP (see Figure 6(a)). The geometric thickness of the object thus varies between 0 and 2 µm. Considering the excitation wavelength of 633 nm, the wavelength of an SPP on a gold/air interface reads *λ*_SPP_ = 605.9 nm, and the wavelength of an SPP on gold/phase object/air structure reads $\lambda_{\text{SPP}}^{\text{obj}}=573.5\;\;nm$ . In geometric optics approximation, the wave passing through unit thickness of the phase object is delayed in phase with respect to the case without the phase object by the difference in wave numbers Δ*k* = 0.586 rad/μm. We utilized FDTD simulations to determine the phase delay Δ*φ* of the SPP wave transmitted through the phase object, defined as the phase of the out-of-plane component of the electric field with the zero phase corresponding to a ray just tangential to the phase object. Next, we utilize this phase delay recorded 500 nm just behind the phase object (along the black dashed line in Figure 6(a)) to estimate the thickness of the phase object as Δ*φ*/Δ*k*. Both the real thickness and the thickness obtained from the phase contrast are compared in Figure 6(b), indicating that the latter represents a good estimate for the former. We expect to achieve improved quantitative performance with optimized imaging protocols.

Structured near field finds numerous fascinating applications also beyond the imaging of phase objects. To begin with, even very fundamental properties of the field including momentum, spin, and angular momentum are the subject of ongoing discussion when considering non-trivial situations such as structured fields in inhomogeneous dispersive media [51]. Here, the complex field patterns can be used to test various definitions of the fundamental field quantities (based on the Abraham or Minkowski approach combined with the kinetic or canonical formalism). These fundamental concepts can be then translated into the formation of chiral fields [52] enabling, e.g., circular dichroism spectroscopy with enhanced sensitivity [53]. Chiral fields or even fields combining angular momentum with radial discontinuity, such as Laguerre–Gauss fields [54, 55], are used in studies of cold Rydberg atoms with the possibility to study the dynamics of quantum many-body systems or get a deeper insight into van der Waals interaction [56, 57]. Recently, a near-field observation of topological transition in all-dielectric higher-order topological insulator metasurfaces has been reported [58]. The full characterization of structured near fields, including both amplitude and phase, is therefore of the utmost importance.

## 4 Conclusions

We have demonstrated a formation of near-field phase patterns with surface plasmon interference devices and characterized the phase patterns both experimentally by SNOM and theoretically by electromagnetic simulations and the analytical model. The patterns can be easily controlled through the polarization of the illumination wave and by dividing the device into the object and reference wave regions. Interestingly, a rich variety of phase patterns can be produced with a single device. Specifically, surface plane waves with controllable propagation vector can be used as a novel tool for imaging and tomography of phase objects such as living cells in the physiological fluid. Further, advanced phase patterns have been demonstrated corresponding to Moiré patterns or simultaneous formation of plane wave regions with different propagation vectors.

## Figures and Tables

**Figure 7:**
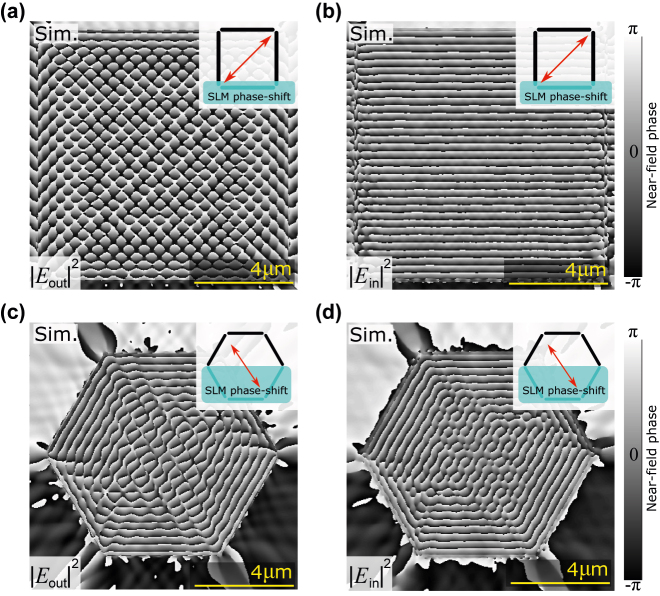
Numerical calculations for individual components of near-field phase distribution on square SPID. Phase distribution of the two components of the electric field (out-of-plane and in-plane) reconstructed from FDTD simulations for two SPIDs (square and hexagonal). (a) and (b) Square SPID, out-of-plane (a) and in-plane (b) components. (c) and (d) Hexagonal SPID, out-of-plane (c) and in-plane (d) components. Insets represent square and hexagonal arrangements of slits with the turquoise patch representing the REF wave area and the red double-sided arrow representing the illumination polarization.

**Figure 8:**
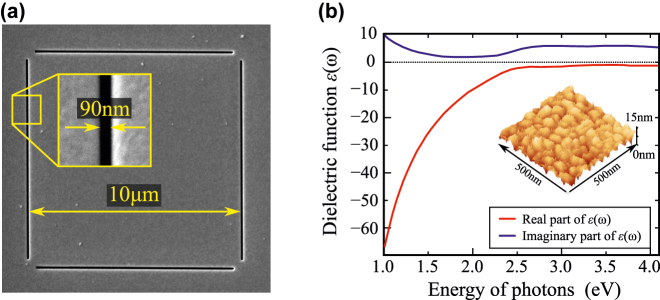
Fabrication of samples. (a) SEM image of the square-like SPID. (b) Ellipsometric measurement of the complex dielectric function for the 200 nm thick gold layer on SiO_2_ substrate deposited by IBS technique. The topography of the deposited layer was measured using AFM revealing maximum surface roughness of less than 15 nm and grain size of approximately 50 nm.

## Appendix A

### A.1 Reconstruction of NF phase from experimental data

The phase of the object (OBJ) wave is reconstructed using phase-shifting digital holography (PSDH). In this approach, the OBJ wave interferes with the reference (REF) wave, the phase of which is actively controlled. The intensity of the resulting hologram is recorded for several values of the phase shift imposed on the REF wave: 0, *π*/2, *π*, and 3*π*/2. We denote the recorded intensities as *I*_1_, *I*_2_, *I*_3_, and *I*_4_, respectively. The phase of the OBJ wave Δ*φ* is then retrieved as

$$\Delta \varphi =\arctan\left(\frac{I_{4}-I_{2}}{I_{1}-I_{3}}\right).$$

More details are provided in Ref. [45]. Both the OBJ and REF waves are formed on the same SPID simply by dedicating some slits or their parts to the OBJ wave and the remaining slits or parts to the REF wave (see Figure 1(c)). To impose the phase shift on the REF wave, we utilize a spatial light modulator (SLM), which enables us to change the phase of illumination light across its wavefront. By the spatial alignment of the illumination beam to the interference structures, we can control the phase of the light impacting different parts of the slits and, therefore, also the phase of the REF wave.

### A.2 Reconstruction of NF phase using FDTD numerical simulations

The intensity of the holograms described in Appendix A.1 is calculated using Ansys Lumerical FDTD, a Maxwell’s equation solver based on a finite-difference time-domain numerical method. All presented results are calculated at a distance of 10 nm above the sample surface. All the material parameters (indices of refraction) used in FDTD simulations are taken from the Ansys Lumerical FDTD internal material database and are based on Handbook of Optical Constants of Solids (ed. E. Palik) [59].

The phase has been retrieved from the intensity distribution of the holograms as described in Appendix A.1. Figure 7 shows an example of reconstructed phase distributions. The simulations allow to perform a vectorial analysis of the field and reconstruct the phase profiles independently for the out-of-plane and in-plane components. A comparison to the experimental phase confirms that our experimental setup is sensitive mostly to the out-of-plane component.

### A.3 Analytical model

The near-field phase distribution within an SPID can also be modeled by purely analytical means. Assuming SPP launched by any slit has a straight wave-front with a uniform amplitude (an SPP analogue to a plane wave in free space), the out-of-plane electric field component of an SPP wave generated by a single slit has the following spatial dependence

$$E_{z}\left(\vec{r}\right)=\frac{\vec{n}\cdot {\vec{E}}_{\text{O}}}{\left|{\vec{E}}_{\text{O}}\right|}e^{\text{i}\beta \left(\vec{n}\cdot \vec{r}\right)},$$

where the dot product between the driving field ${\vec{E}}_{0}$ and the unit normal vector $\vec{n}$ (perpendicular to the slit) determines the SPP amplitude. The SPP wavelength *λ*_SPP_ and propagation length *L*_SPP_ is naturally dictated by the complex SPP wavevector

$$\beta =\beta^{\prime }+i\beta^{\prime \prime }=\frac{2\pi }{\lambda_{\text{SPP}}}+i\frac{1}{L_{\text{SPP}}}.$$

The task of calculating the phase distribution within an SPID now amounts to adding the field profiles of all SPP waves that it generates, while maintaining the distinction between OBJ and REF waves. Denoting *φ*_O_(*x*, *y*) and *φ*_R_(*x*, *y*) the phase distributions of the OBJ and REF waves ${\vec{E}}_{\text{O}}\left(x,y\right)$ and ${\vec{E}}_{\text{R}}\left(x,y\right)$ , the digital holography technique enables us to compute their difference as

$$\varphi_{\text{O}}\left(x,y\right)-\varphi_{\text{R}}\left(x,y\right)=\arctan\left[\frac{Im\left\{E_{\text{R}}^{*}\left(x,y\right)\cdot E_{\text{O}}\left(x,y\right)\right\}}{Re\left\{E_{\text{R}}^{*}\left(x,y\right)\cdot E_{\text{O}}\left(x,y\right)\right\}}\right].$$

In the case of the square SPID analyzed in Figure 2(d), the driving field polarization and SLM zones were chosen in such a way that the OBJ and REF have the following form

$$\begin{array}{ll} E_{\text{O}}\left(x,y\right) & =A{\text{e}}^{+i\beta^{\prime }x}{\text{e}}^{-\beta^{\prime \prime }x}-A{\text{e}}^{-i\beta^{\prime }x}{\text{e}}^{+\beta^{\prime \prime }x}\\ & \;+A{\text{e}}^{+i\beta^{\prime }y}{\text{e}}^{-\beta^{\prime \prime }y},\\ E_{\text{R}}\left(x,y\right) & =-Ae^{-i\beta^{\prime }y}{\text{e}}^{+\beta^{\prime \prime }y}. \end{array}$$

The signs both in front of and inside the exponentials are clearly the result of the dot product between the respective normal vectors of the slits and the driving field vector/spatial vector. The phase distribution for the hexagonal SPID was obtained in a similar manner, using the same values for the SPP wavelength and the propagation length, i.e., *λ*_SPP_ = 605 nm and *L*_SPP_ = 10 μm. We should note that this analytical calculation is generally valid only in the centre of the SPID, where our assumptions about the shape and uniformity of the SPP wave-front roughly hold.

### A.4 Fabrication of samples

The SPIDs used in the experiment consist of a fused silica (SiO_2_) substrate that was polished on both sides to a resulting average surface roughness of less than 15 nm (determined by atomic force microscopy, AFM, NT-MDT Ntegra). Subsequently, a layer of gold was deposited on the polished substrate using the ion beam sputtering (IBS) in a high vacuum. The thickness of the deposited layer was approximately 200 nm. Between the substrate itself and the gold layer, a 3 nm adhesion layer of titanium was deposited (with a negligible influence on the optical properties of the sample).

The slits serving as the SPP sources were fabricated using focused ion beam (FIB) milling in dual beam scanning electron microscope (SEM) Tescan Lyra3 equipped with gallium liquid metal ion source. The slits were approximately 10 µm long and 90–100 nm wide, and their depth extended below the gold layer (i.e., more than 200 nm). Figure 8(a) shows the SEM image of the slits, which has been utilized to set the model for FDTD simulations (see Figure 1(a) and (b)).

### A.5 Experimental setup

The experimental setup is shown in Figure 9. The intensity of the near field was obtained using an aperture-type scanning near-field optical microscope (a-SNOM) made by NT-MDT (NTEGRA Solaris), mounted on an Olympus IX71 inverted optical microscope, which was used to introduce and focus the laser beam onto the sample. The used commercial SNOM probe is made of single-mode optical fibre Nufern 460HP, where the tip of the probe is metal-coated by a 70 nm thin film of aluminium with a 20 nm thin Cr sublayer. The distance between the SNOM probe and the surface of the gold layer was in the nanometer range, and the diameter of the probe aperture was in the interval (100 ± 30) nm. A red He–Ne laser with the wavelength of 632.8 nm and output power of 20 mW was used to illuminate the sample. The laser beam was focused on the sample by the objective with a 60× magnification (numerical aperture NA = 0.6). The size of the laser spot on the sample was approximately 20 µm.

The laser beam light passes through a linear polarizer (P0), a beam splitter (BS), and an expander (Exp) where the beam diameter is increased by a factor of 10. The expanded laser beam impinges the spatial light modulator (SLM), which imposes the desired phase distribution on the parts of the wavefront used to excite the OBJ and REF waves. The beam reflected from SLM goes back through Exp and BS. After that, the laser beam is guided by the mirrors and focused on the sample by the objective. The SLM (Hamamatsu) used in the experiments has a display size of (15.8 × 12.0) mm^2^ and a resolution of (800 × 600) pixel^2^.
